# Quorum Decision-Making in Foraging Fish Shoals

**DOI:** 10.1371/journal.pone.0032411

**Published:** 2012-03-07

**Authors:** Ashley J. W. Ward, Jens Krause, David J. T. Sumpter

**Affiliations:** 1 Department of Biology, University of Leicester, Leicester, United Kingdom; 2 School of Biological Sciences, University of Sydney, Sydney, Australia; 3 Leibniz Institute of Freshwater Ecology and Inland Fisheries, Berlin, Germany; 4 Mathematics Department, Uppsala University, Uppsala, Sweden; Arizona State University, United States of America

## Abstract

Quorum responses provide a means for group-living animals to integrate and filter disparate social information to produce accurate and coherent group decisions. A quorum response may be defined as a steep increase in the probability of group members performing a given behaviour once a threshold minimum number of their group mates already performing that behaviour is exceeded. In a previous study we reported the use of a quorum response in group decision-making of threespine sticklebacks (*Gasterosteus aculeatus*) under a simulated predation threat. Here we examine the use of quorum responses by shoals of sticklebacks in first locating and then leaving a foraging patch. We show that a quorum rule explains movement decisions by threespine sticklebacks toward and then away from a food patch. Following both to and from a food patch occurred when a threshold number of initiators was exceeded, with the threshold being determined by the group size.

## Introduction

Group living animals have access to both private information that they collect themselves and social information gained from observing those around them [1], [2], [3]. The use of social cues potentially allows individuals to access large amounts of information at low cost and hence their use is widespread and occurs in a variety of different contexts, including foraging [4], [5], navigation [6] and under predation threat [7]. The use of social information may often increase foraging efficiency, especially where food is patchily distributed [8], [9]. But while the use of social information potentially allows an individual to exploit its local environment more effectively, simple acceptance or ‘blind copying’ of inaccurate social information can reduce efficiency [10], [11], [12]. Furthermore, animals must frequently make trade-offs between conflicting demands, for example in deciding whether to forego a food patch so as to remain with a departing group of conspecifics [13], [14], [15]. If an individual remains at the food patch when its group mates move off, it pays a cost in terms of losing the benefits of sociality to do so. However, if it responds to social cues and leaves the food patch with the rest of the group, it misses out on a valuable foraging opportunity.

If individuals have simultaneous access to both private and social information, the question is then how to achieve a good balance between these. In studies of social insects, where social and kin structure removes the dilemma of having food finds parasitized by others, theoretical and empirical studies have concentrated on mechanisms for achieving this balance [16], [17], [18]. Individuals that have found food signal its location to others and a positive feedback loop ensues whereby an increasing number of nestmates are recruited to the food. As a result, the colony can find the shortest route to a food source [19] and direct their foragers to the better of two available sources [20], [21].

Recent studies of gregarious insect and free forming vertebrate groups have found that positive feedback also plays an important role in their collective decision-making [22], [23], [24], [25]. The responses to conspecifics in these cases are often quorum responses, in which an animal's probability of exhibiting a behaviour is a sharply non-linear function of the number of other individuals already performing this behaviour [26]. Quorum rules do not require active signalling between individuals but can be mediated through behavioural cues, and are thus consistent with the concept in producer-scrounger models that some individuals parasitize food discoveries. Simply by watching the behaviour of others, individuals are able to increase their own decision-making accuracy [26], [27], [28], [29], [30].

In this paper, we study the mechanisms used in the movement decisions of sticklebacks in a putative social foraging context. We used remote controlled replica sticklebacks to initiate movements towards a food patch and, later, away from the patch. We subsequently examine whether the behaviour of live sticklebacks is consistent with quorum responses.

## Methods

### Ethics statement

All experiments were conducted in accordance with guidelines for animal research provided by the University of Leicester and the UK Home Office. Specific approval for this research was not required since it involved purely observational behavioural experiments. Collection permits were not required for the capturing of sticklebacks using hand nets at our collection point (the River Welland, Leicestershire (52°30′35″N; 0°53′18″W)). Access to the collection site is provided by a public right of way and the location is not privately-owned or protected in any way. Field collection of fishes did not involve or affect any endangered or protected species.

### Study animals

We collected three-spined sticklebacks from the River Welland in October 2003. All fish used were juveniles measuring 30±4 mm. The fish were maintained thereafter in groups of 20 fish in each of 20 40 L aquaria at the University of Leicester, UK at 12°C with a 12 hours light, 12 hours dark regime and fed daily with defrosted frozen bloodworms. Following the completion of the experiments, the fish were retained in the aquarium facility at the University of Leicester.

### Experimental protocol

**Figure 1:**
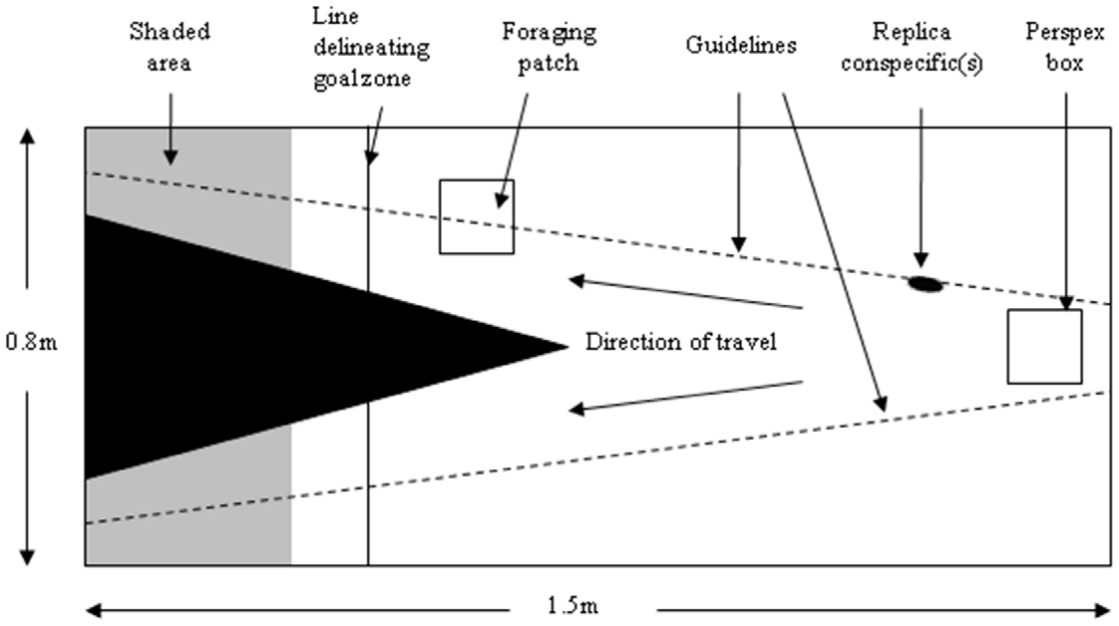
Experimental set-up.

To examine decision-making in groups of fish, we constructed an experimental arena, akin to a Y-maze, that offered a choice of two identical refuges, both equidistant from a starting point (Fig. 1). Within the arena, two monofilament lines were extended across the arena from positions just behind a starting point to the refuges at the opposite end of the arena. One monofilament line was placed on each side of the starting point. The refuges were constructed by shading a 30 cm portion of the aquarium. Replica stickleback(s) (see [22] for details of the construction of the replicas) could then be mounted on these lines and pulled along them by a remote-controlled electric motor at a speed of 4 cm/second from the starting point to one of the refuges. This speed was determined to produce the strongest following response during pilot trials.

Experimental fish were fed two hours prior to their use in trials in an effort to standardise feeding motivation of experimental subjects across trials. At the outset of each trial, live test fish were added to a clear, bottomless Perspex box measuring 15×12×20 cm (l×w×h) positioned at the starting point. Either 0, 1 or 2 replica fish were positioned alongside the box at a distance of 8 cm from it. Following their introduction, the test fish were allowed to acclimatise for 5 minutes before the box was raised, releasing the fish. Simultaneously, the electric motor was started causing the replica(s) to move off in the direction of one of the refuges at a speed of 4 cm/s. Along the route of the replica fish, we placed a simulated food patch, an 8×8×0.5 cm (1×w×h) clear plastic dish with lid containing >100 live bloodworms. Bloodworms (Chironimid larvae) are a favoured food of sticklebacks, moreover the bright red colouration is intensely attractive to sticklebacks, which have a powerful sensory receiver bias for the colour red. In pilot tests we determined that when solitary fish located such a food patch in their home aquarium, they vigorously attempted to eat the worms. Only 7% (1 in 16) of sticklebacks left the patch within the first two minutes following their arrival, despite being unable to either smell or eat the worms. For this reason, we feel confident in asserting that the experimental subjects treat the plastic dish as an attractive foraging patch, especially in the short term relevant to the current experiment. When the replica fish reached the food patch, it paused for 30 seconds before moving off again to the refuge. The experiments continued until all fish had entered the shaded goal zones or refuges, or 60 seconds had elapsed since the replica fish moved off from the food patch, whichever came first. The maximum total time that the live fish could spend at the foraging patch during the experiment was therefore less than the two minute period used in the pilot trials. The side at which the replica individual(s) were presented was randomized. All trials were filmed from above. We manipulated the number of replica fish between trials, using either 0, 1 or 2 replica fish. In addition, we used test fish group sizes of 1, 2, 4 and 8. 20 replicates were performed for each combination of group size and replica number. Each fish was used only once. Treatment and trial order were randomised.

To compare the numbers of fish that followed the replica leaders between treatments we used a χ^2^ test of independence, comparing the number of fish that went to the food patch against the number that did not go to the food patch in the presence of 0, 1 and 2 leaders for each group size, and the number of fish that left the food patch against the number of fish that did not leave the food patch, again in the presence of 0, 1 or 2 leaders for each group size.

### Model

The two stages of the experiment, to the food patch and away from the food patch, were modelled separately. For the approach to the food patch we adopt the same model and the same parameters as measured in Ward et al. [22] in the absence of a food patch, thus providing an independent test of the parameters measured in that work.

#### Approaching a food patch

The model is based on the hypothesis that the propensity for taking the maze branch with food increases as a function of the number of individuals that have gone towards the food and decreases with the number that have either gone away from food or remain uncommitted. In particular, this is a steep sigmoidal function such that the probability of moving in a particular direction increases sharply with the number of other fish which have recently moved in that direction. Specifically, the probability of an uncommitted individual going towards food on time step *t*+1 is
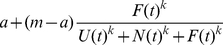
(1)where *U(t)* is the number of uncommitted individuals at time *t*; and *F*(t) and *N*(t) are, respectively, the total number of individuals that have taken the branch containing and not containing food [22].

The three parameters—*a* which is the accept rate in the absence of conspecifics, *m* which is the maximum probability per time step of committing to a decision and *k* which is the steepness of response—determine the shape of this response. The setup of the current experiment is similar to that of Ward et al. [22] and uses fish from the same population (albeit different individuals), but this time includes a food patch. Therefore, the parameters—*a* = 0.0078, *m* = 0.25 and *k* = 3.2—were set to be those estimated from our earlier experimental work in the absence of a food patch [22] and are thus established independently from the current experiment. The fact that *k* is greater than one indicates that the response to other fish is indeed a steeply increasing quorum-like response [26]. The parameters determine the probability per time step of going to the food and thus depend on the time step of the model. In Ward et. al [22] we included a parameter *T* in the model, which determined how long back in time a focal fish would monitor left and right movements of conspecifics. In that paper (and in an analysis of the results of the current experiment) we found that the model fit best when all previous movements were integrated, and on this basis we omit a *T* in our current description.

**Figure 2:**
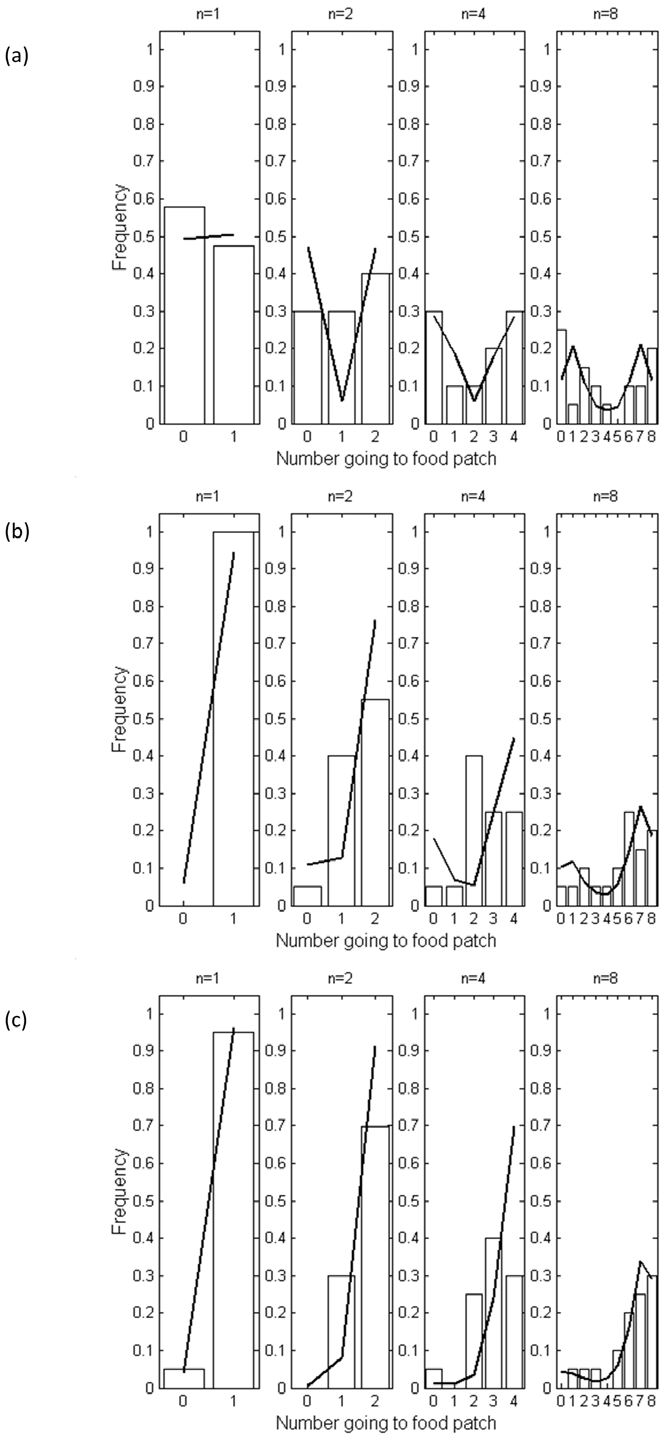
Graphs show the distribution of the number of fish that went to the food patch for each group size (*n*) for (a) no leader, (b) one leader and (c) two leaders. Model predictions based on the outcomes of 10,000 runs of the quorum response model are indicated by a solid line in each case.

The commitment probability per time step for taking the branch without food is
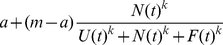
(2)Note that the spontaneous probability of moving towards food is the same as that for moving away from food. This reflects the fact that, without the replica leader(s), the test fish were unable to detect which branch contained the foraging patch (see results and figure 2a in particular). Indeed, the only difference initially between the food and non-food branches is generated by the replicas. We set *N*(0) = 0 and *F*(0) equal to the number of replica fish travelling to the food and *U*(0) equal to the group size of the test fish.

#### Leaving a food patch

At this stage of the experiment the fish choose between leaving or staying at the food patch. We thus set the probability of an individual leaving food on time step *t*+1 as
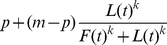
(3)where F*(t)* is the number of individuals at the food patch and *L*(t) is the cumulative number of individuals which have left the food patch. In accordance with the experiment, we set the initial values *F*(0) to be the number of individuals which travelled to the food patch and *L*(0) to be the number of replica fish leaving the food patch observed in the experimental trials. Independent of the number of fish at the start of the experiment, we group together all trials in terms of *F*(0) independent of the initial group size. This grouping assumes that once at the food patch, the fish behaviour does not depend on their group size before travelling there.

Since we are interested in measuring how many fish were left on the food patch at the end of the trial, we need to define a time step for our simulation. The Ward et al. [22] experiments are consistent with setting the length of one time step to be one second, although no explicit attempt is made to measure the time taken for individuals to make a decision. In order to fit the current model, we note that in the case of a single fish with no leader, at the end of the experiment in 83% of trials the fish is still on the food. Solving 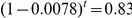 (where as above *a = 0.0078*) tells us that by running the simulation for 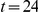 time steps we can reproduce the experimental result for a single fish with no leader. Since the experiments are 60 seconds long, this means that one time step of the simulation is 2.5 seconds long. Consequently, individuals on a food patch have a lower rate of leaving than individuals at the release point.

## Results

We performed model simulations in order to compare the model to the data. For the approach to the food patch all parameters were already measured from previous work (see above) and we simply compared 10,000 runs of the simulation to the outcome of the experiments.

The replica leaders significantly increased the visitation of food patches. Figure 2 presents a frequency distribution of the number of individuals travelling to the food patch for each of the treatments. In the absence of replica leader fish there is no evidence to reject the null hypothesis that the fish choose the left or right channel at random (Sign test: Group size 1, n = 20, p = 1; group size 2, n = 20, p = 0.79; group size 4, n = 20, p = 0.82; group size 8, n = 20, p = 0.65). For group sizes of 4 and 8 individuals, the distribution of the number going to the food in the absence of a leader is U-shaped and inconsistent with independent decision-making (Independent decision-making would lead to a binomial distribution for which we test, group size 4: 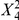 = 42.1, p<0.001; Group size 8: 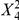 = 519.8, p<0.001). This observation supports our earlier work on this species in a putative predation context that some form of non-linear feedback determines the movement decisions of the fish.

The presence of replica leaders generally increases the frequency with which test fish move to the food patch for all group size treatments compared to when there is no replica leader (see Table 1). The presence of two leader replicas increases the frequency with which test fish move to the food patch compared to when there is one replica leader only for the largest group size of 8. Again, these results are inconsistent with independent decision-making, which would predict that proportion of individuals following the leader would be the same in all treatments. In summary, the effect of leaders is more pronounced for singletons and when there are more leaders.

**Table 1:** The fraction of all test fish that followed replica leaders towards the food patch.

		Initial group size			
		1	2	4	8
Leaders	0	9/20	22/40	42/80	75/160
	1 *0v.1*	20/20 *P<0.01*	30/40 *P = 0.061*	52/80 *P = 0.1*	105/160 *P<0.01*
	2 *0v.2 1v.2*	19/20 *P<0.01 P = 0.31*	34/40 *P<0.01 P = 0.26*	58/80 *P<0.01 P = 0.31*	123/160 *P<0.01 P = 0.03*

The numerator is the number of test fish going to the food patch across all trials, the denominator is the number of test fish that started the trial. Tests to compare the number of fish that went to the food patch in the presence of 0 versus 1 leader, 0 versus 2 leaders and 1 versus 2 leaders between were carried out using a χ^2^ test of independence. Two-tailed *P* values are presented for comparisons at each group size.

The solid line in figure 2 compares the quorum model with the data. The model prediction was not significantly different from the experimental outcome in any of leader/group size treatment combinations. The data is not however consistent with a non-quorum like model of decision-making, i.e. with *k* = 1. In particular, outcome for groups of size 4 was significantly different in the model than in the data (one leader 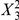 = 9.9 one leader 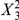 = 8.7, both *P*>0.05). For groups of size 8 the *k* = 1 was not significantly different from the data, but provided a poorer fit than the quorum model established in earlier work [22].

**Figure 3:**
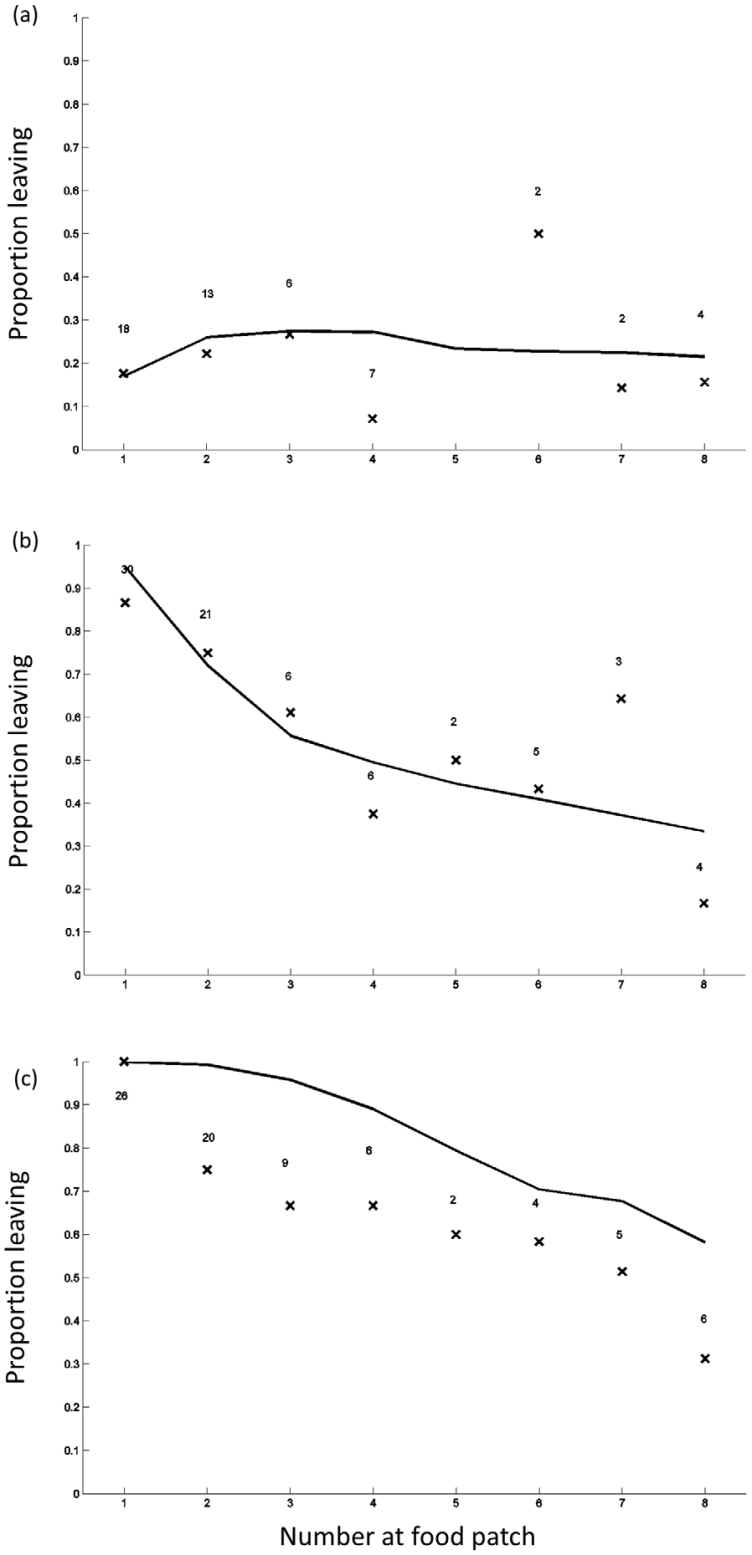
Proportion of fish at the food patch that left the food patch immediately following the departure of the replica leader(s) are marked by crosses for (a) no leaders (b) one leader and (c) two leaders. The number directly above the crosses is the number of observations in which this number of fish was observed at the food patch. Model predictions based on a quorum response are indicated by the solid line.

Movements away from the food patch were also significantly greater in the presence of a leader, or leaders, and decreased with the initial group size (Table 2) unless there was no leader, in which case the probability of leaving was independent of initial group size. However, the initial group size does not provide a complete picture of decisions to leave the food patch, since the group arriving at a food patch was usually smaller than the test group size. Figure 3 presents the proportion leaving as a function of group size at the food patch, with all the data with different initial group sizes pooled. Again, movements away from the patch increase with number of leaders and decrease with group size (again with an exception in the case of no leader). The tendency of individuals to follow a single leader from the food patch was lower than their tendency to follow from the initial start position in group sizes of 4 and 8, while the tendency to follow two leaders was fairly consistent across the two contexts in smaller group sizes but lower from the food patch for fish in a group size of eight (Table 3).

**Table 2:** The fraction of all test fish that followed replica leaders away from the food patch according to their initial group size.

		Initial group size			
		1	2	4	8
Leaders	0	1/9	3/22	1/42	10/75
	1 *0v.1*	18/20 *P<0.01*	23/30 *P<0.01*	26/52 *P<0.01*	37/105 *P<0.01*
	2 *0v.2 1v.2*	19/19 *P<0.01 P = 0.16*	27/34 *P<0.01 P = 0.79*	39/58 *P<0.01 P = 0.07*	58/123 *P<0.01 P = 0.07*

The numerator is the number of test fish leaving the food patch across all trials, the denominator is the number of test fish that went to the food patch. Tests to compare the number of fish that left the food patch in the presence of 0 versus 1 leader, 0 versus 2 leaders and 1 versus 2 leaders between were carried out using a χ^2^ test of independence. Two-tailed *P* values are presented for comparisons at each group size.

**Table 3:** The fraction of all test fish that followed replica leaders away from the food patch according to the group size at the food patch, rather than the group size that initially started the trial.

		Group size			
		1	2	4	8
Leaders	1	26/30 *P = 0.14*	30/42 *P = 0.81*	9/24 *P = 0.02*	4/32 *P<0.01*
	2	26/26 *P = 0.43*	30/40 *P = 0.26*	16/24 *P = 0.61*	15/48 *P<0.01*

The numerator is the number of test fish leaving the food patch across all trials, the denominator is the number of test fish that went to the food patch. Tests to compare the number of fish that went to the food patch against the number of fish that left the food patch in the presence of 1 leader and in the presence of 2 leaders were carried out using a χ^2^ test of independence. Two-tailed *P* values are presented for comparisons at each group size.

Overall the model fits the data well, following the pattern of decreasing leaving tendency with increasing group size. The one quantitative discrepancy is that the model underestimates the tendency of the fish to leave when there are two leaders.

## Discussion

The probability of following both to and from a food patch was a function of group size and of the number of initiators. The tendency of test fish to follow replica conspecifics decreased with increasing group size. Furthermore, the response to the replicas and to other group members was consistent with a quorum rule. Parameters estimated from earlier data sets proved sufficient to reproduce the results of our current experiment, hence fish behaviour in these contexts is consistent with the same quorum rule employed under predation risk [22] and in distinguishing phenotypic differences [30], which provides support for the idea that quorums are a general mechanism for animal decision-making across a wide range of contexts. We found one discrepancy between the model and the data, that two leaders were less likely than predicted to lead a group away from food. It is possible that slightly different functional forms of equations 1 to 3, for example using the logistic equation, could provide a better fit to the data (e.g. [31]). Nonetheless, the approach we have presented here is powerful in that we can make reasonable quantitative predictions about the rules governing the fish's behaviour.

The departure of individuals from a group conveys information to the other group members. The departing individual or individuals may do so in response to a decrease in patch profitability or in response to some other factor, for instance the approach of a predator. The greater tendency of fish in small groups to respond to the departure of a single replica or group member may reflect a foraging *versus* safety trade-off, since individuals in smaller groups are at greater risk of predation than those in larger groups and are more attuned to potential threat cues [32], [33], [34], [35]. The responsiveness to individual departures potentially leaves fish in small groups prone to errors. By contrast, fish in larger groups are less responsive to the single departures and are thus at less risk of errors. However, the departure of at least two individuals from the group is used as a cue by other group members that the departing individuals possess some additional information and triggers a much stronger following response. As a result, the quorum rule allows the group to remains cohesive, while exploiting the information possessed by those moving away from food. These dual properties of ensuring group cohesion while increasing decision-making accuracy make the quorum rule a powerful yet simple mechanism underpinning collective decision-making [26].

One important question is how this essentially mechanistic quorum response model might be integrated with functional models of social foraging, which predict how animals make strategic, economic foraging decisions in a dynamic environment in the presence of other foragers, where resource availability is modified by those other foragers [36]. Producer-scrounger models are an important category of social foraging model. These generally predict that a population will reach a balance between producers, who look for food, and scroungers, who watch the producers and scrounge their discoveries [37], [38], [39], [40]. A key prediction of producer scrounger models is that joining behaviour increases when food patches are larger and richer. This prediction holds also when animals forage according to a quorum rule as the quorum response can automatically tune joining rate to the patchiness of the food [41]. Although in general there is no reason why over time an individual cannot be both a producer and a scrounger [42], [43], there is a growing appreciation that rather than being solely strategic, the adoption of producer or scrounger roles may be linked in to an individual's behavioural phenotype [44]. This raises the intriguing question of how individuals with different personality types might apply and respond to quorum rules in social foraging and indeed other contexts. We argue that the application of a mechanistic approach to social foraging does not replace or supercede functional models of social foraging. Indeed, the use of a combined approach provides the most powerful framework for better understanding the behaviour of animals. The challenge is to meld this proximate understanding more closely with functional explanations of how individuals integrate private and social information when foraging [45], [46].

While the quorum rule appears to provide a relatively robust rule for tuning joining behaviour to particular environments, there is some evidence that the individuals change their response to different situations. In particular, the parameters of the quorum response may change depending on environmental context. Our experimental results show that individuals in larger groups of 4 and 8 had a reduced tendency to follow a single replica leader from a food patch than from their initial starting position, where there were no apparent resources. By contrast, the departure of two replica leaders from the food patch produced a stronger following response, and one that was similar to the following response from the initial starting position for solitary individuals and those in groups of 2 and 4. Fish in a group size of 8 were less likely to follow two replica leaders from a food patch than from the initial, resource-free patch. These results suggest that the quorum response can be modified according to group members' private information regarding the availability of resources. Such tuning of quorum rules may be a common feature in decision-making. For example, *Temnothorax* ants, which use a quorum to decide whether to commit to a new nest site during emigration, adjust various parameters of their quorum response as a result of differences in the urgency of their emigration [47], [48]. The question of how the parameters relating to such decision rules change across a range of ecological conditions is an obvious vital next step to understanding the evolution of social responses to the environment.
